# Supplementary figures and images for: Gene and genome-centric analyses of koala and wombat fecal microbiomes point to metabolic specialization for Eucalyptus digestion
Source: PeerJ. 2017 Nov 16;5:e4075. doi: 10.7717/peerj.4075 (PMC5697889; doi:10.7717/peerj.4075)

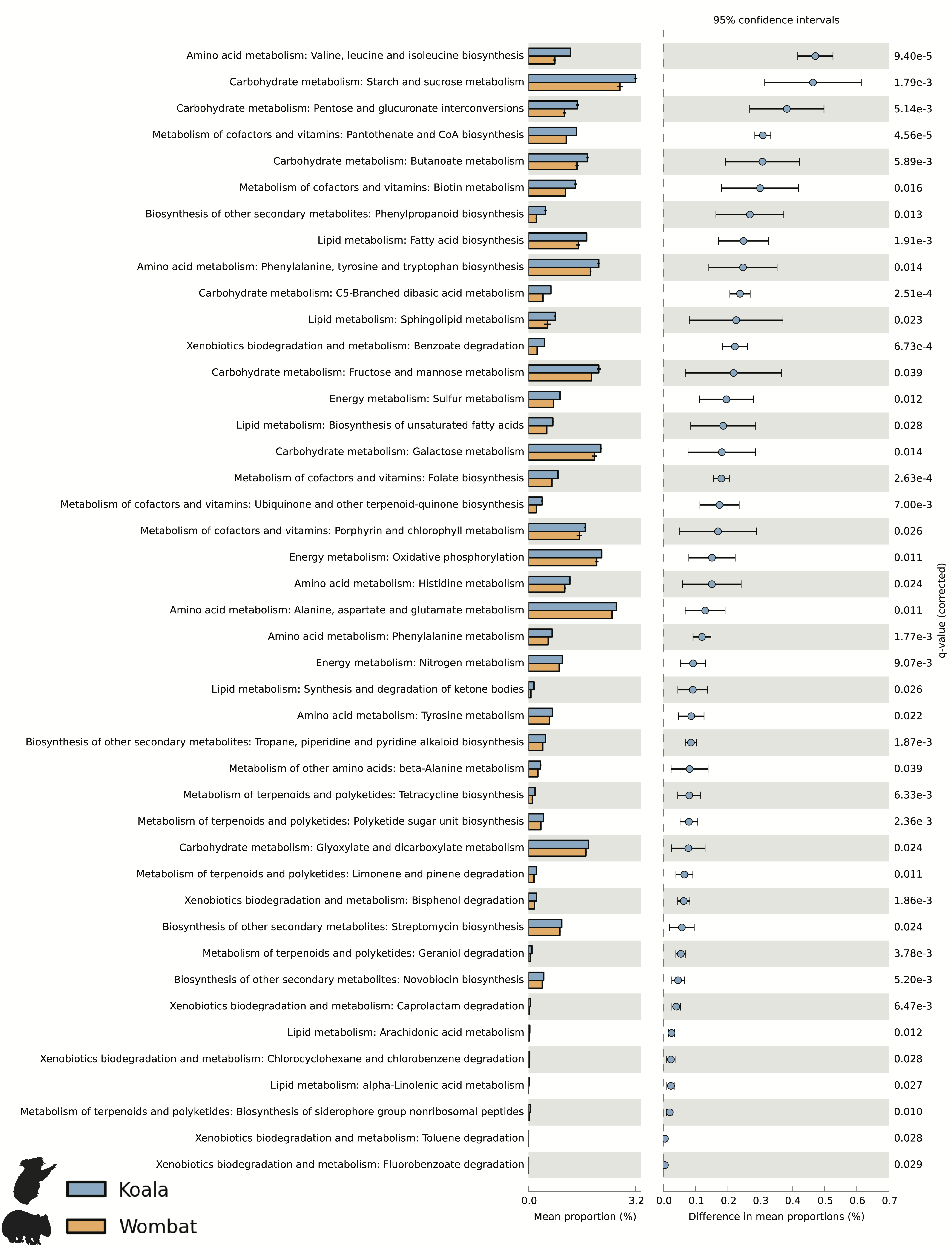

Supplement: Figure S1 — Predicted CDSs from each marsupial co-assembly were annotated based on KEGG Orthology (KO) using the KEGG Automatic Annotation Server (Moriya et al., 2007). KO annotations were weighted by average coverage of associated contigs per sample time-point and normalized to overall coverage of all CDSs. Annotations were assigned to all corresponding KEGG pathways according to the KEGG metabolism hierarchy (1.1–1.11), and pathways were filtered to exclude those containing fewer than three KOs. Relative cumulative prevalence of KEGG metabolic pathways from each marsupial host were then compared using STAMP (Parks et al., 2014). Differential pathways were identified based on significant difference between datasets (q < 0.05, Benjamini-Hochberg FDR correction) and ranked by effect size (difference in mean proportion). [file peerj-05-4075-s001.png]

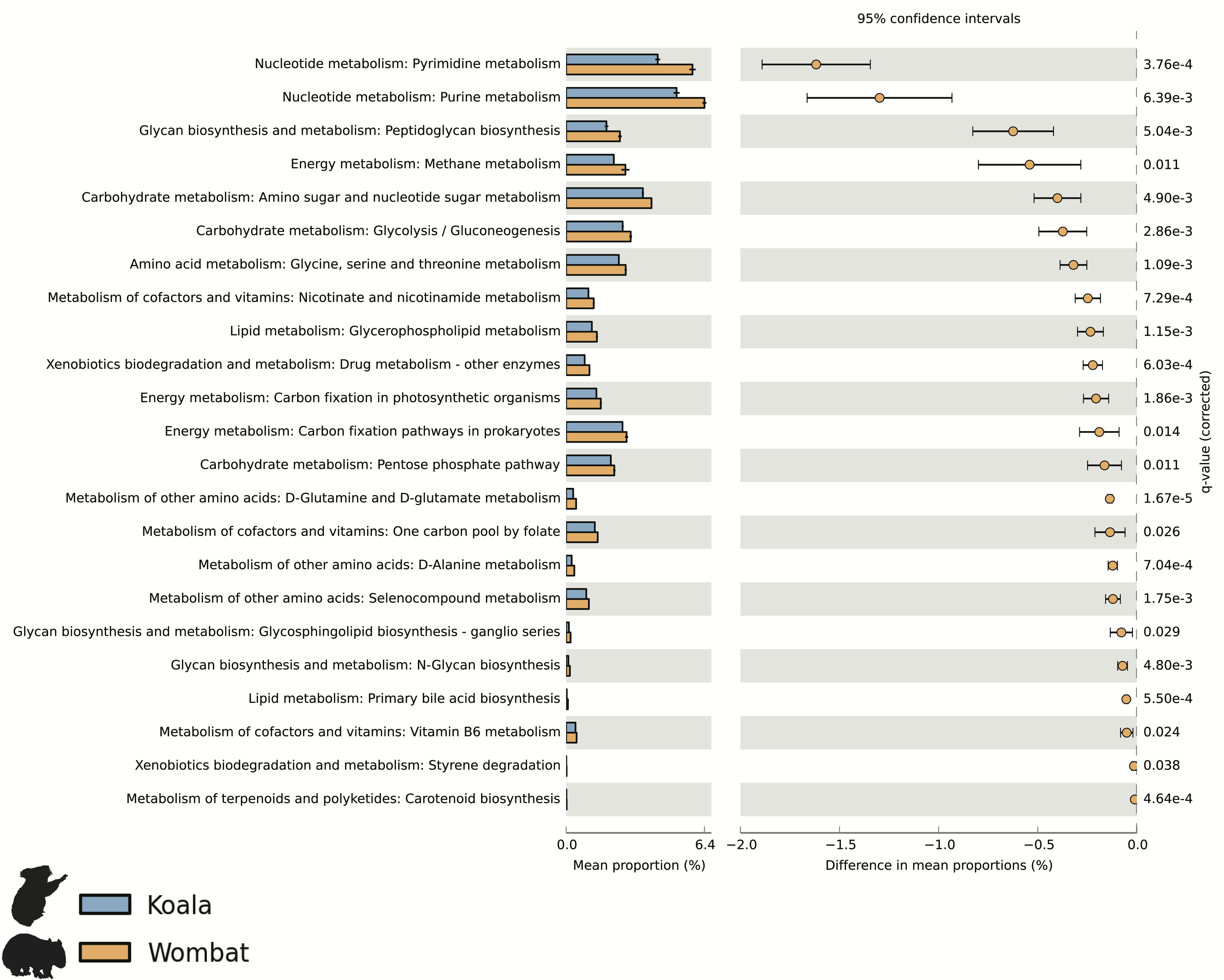

Supplement: Figure S2 — Predicted CDSs from each marsupial co-assembly were annotated based on KEGG Orthology (KO) using the KEGG Automatic Annotation Server. KO annotations were weighted by average coverage of associated contigs per sample time-point and normalized to overall coverage of all CDSs. Annotations were assigned to all corresponding KEGG pathways according to the KEGG metabolism hierarchy (1.1–1.11), and pathways were filtered to exclude those containing fewer than three KOs. Relative cumulative prevalence of KEGG metabolic pathways from each marsupial host were then compared using STAMP (Parks et al., 2014). Differential pathways were identified based on significant difference between datasets (q < 0.05, Benjamini-Hochberg FDR correction) and ranked by effect size (difference in mean proportion). [file peerj-05-4075-s002.png]

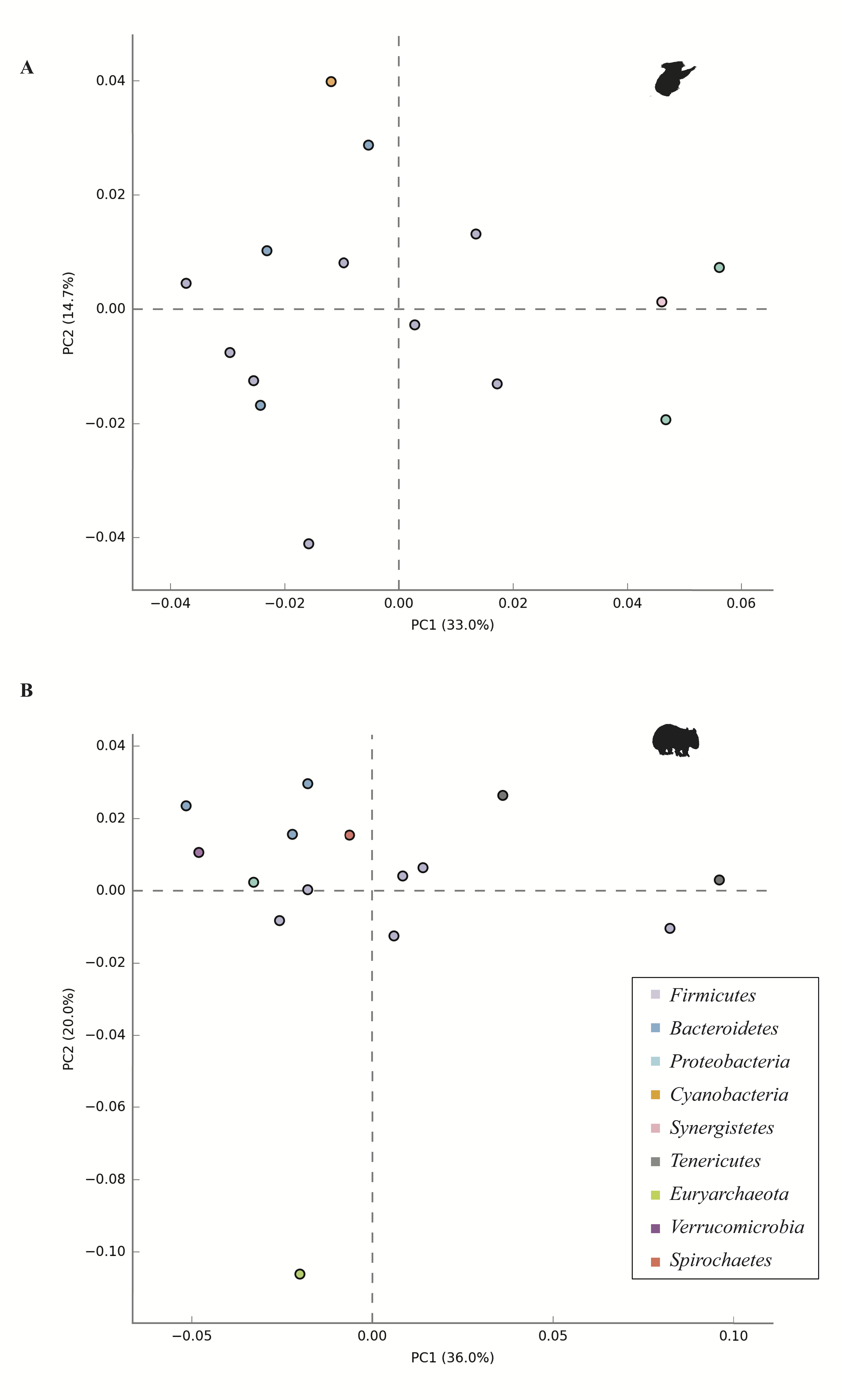

Supplement: Figure S3 — The 15 most abundant quality population genomes from the (A) koala and (B) wombat communities were annotated based on KEGG Orthology (KO) (Moriya et al., 2007). Annotations were assigned to all corresponding KEGG pathways according to the KEGG metabolism hierarchy (1.1–1.11), and pathways were filtered to exclude those containing fewer than three KOs. Principal component analysis was used to differentiate among populations by treating each genome as a collection of metabolic pathways with variations in relative abundance: thus, genomes with more similar distributions of metabolic potential appear closer in space. Circles corresponding to plotted genomes are colored by phylum. [file peerj-05-4075-s003.png]
