# Supplementary material for: Gene and genome-centric analyses of koala and wombat fecal microbiomes point to metabolic specialization for Eucalyptus digestion
Source: PeerJ. 2017 Nov 16;5:e4075. doi: 10.7717/peerj.4075 (PMC5697889; doi:10.7717/peerj.4075)
Supplement: Table S1 — Fecal samples representing multiple time-points from a captive koala and captive wombat were sequenced, generating a total of 90.7 Gb and 22.0 Gb of raw data, respectively. [file peerj-05-4075-s004.docx]

|  | **Time-**  **points** | **Library prep** | **Sequencing platform** | **Lanes per sample** | **Paired-end read length** | **Raw reads** | **Mbp** |
| --- | --- | --- | --- | --- | --- | --- | --- |
| Wombat | 5 | Nextera | Illumina HiSeq 1000 | 1/5 lane | 2x 100 bp | 34,600,000 – 51,600,000 | 3460 – 5160 |
| Koala | 2 | Nextera | Illumina  HiSeq 2000 | 1/3 lane | 2x 150  bp | 71,200,000 – 100,000,000 | 10,679 – 15,003 |
|  | 1 | TruSeq |  | 1 lane |  | 433,800,000 | 65,065 |
