# Supplementary material for: Gene and genome-centric analyses of koala and wombat fecal microbiomes point to metabolic specialization for Eucalyptus digestion
Source: PeerJ. 2017 Nov 16;5:e4075. doi: 10.7717/peerj.4075 (PMC5697889; doi:10.7717/peerj.4075)
Supplement: Table S2 — Shotgun reads corresponding to 16S rRNA sequences were identified with HMMs and mapped to the Greengenes 97% database (McDonald et al., 2012), and the resulting community makeup was assessed at each taxonomic level (including unmapped reads). Lineages with less than 0.05% of corresponding reads were grouped and reported as “Other” (if the resulting group represented >0.05% of the community). Here, k, represents kingdom; p, represents phylum; c, represents class; o, represents order; f, represents family; and g, represents genus. [file peerj-05-4075-s005.docx]

| **Taxonomy** | **Koala_1** | **Koala_2** | **Koala_3** |
| --- | --- | --- | --- |
| k__*Bacteria* | 81.83 | 79.13 | 77­­.16 |
| p__*Bacteroidetes* | 21.21 | 13.73 | 16.49 |
| c__*Bacteroidia* | 21.19 | 13.65 | 16.44 |
| o__*Bacteroidales* | 21.19 | 13.65 | 16.44 |
| f__*Bacteroidaceae* | 7.79 | 5.16 | 4.07 |
| g__*Bacteroides* | 7.78 | 5.16 | 4.06 |
| f__*Porphyromonadaceae* | 0.80 | 0.81 | 1.29 |
| g__*Parabacteroides* | 0.76 | 0.81 | 1.19 |
| f__*Rikenellaceae* | 1.40 | 0.33 | 1.49 |
| g__unclassified | 1.27 | 0.33 | 1.30 |
| f__*S24-7* | 10.55 | 5.63 | 8.78 |
| g_*_*unclassified | 10.55 | 5.63 | 8.78 |
| f__unclassified | 0.48 | 1.54 | 0.53 |
| p__*Cyanobacteria* | 5.92 | 6.00 | 9.00 |
| c__*4C0d-2* | 5.92 | 5.99 | 9.00 |
| o__*YS2* | 5.92 | 5.98 | 9.00 |
| f__unclassified | 5.92 | 5.98 | 9.00 |
| p__*Firmicutes* | 33.79 | 29.18 | 39.67 |
| c__*Clostridia* | 33.06 | 28.96 | 38.86 |
| o__*Clostridiales* | 33.05 | 28.93 | 38.86 |
| f__*Clostridiaceae* | 0.52 | 0.32 | 1.05 |
| g__*Clostridium* | 0.51 | 0.32 | 1.03 |
| f__*Lachnospiraceae* | 8.21 | 2.84 | 5.83 |
| g__*Blautia* | 0.84 | 0.12 | 0.44 |
| g__unclassified | 6.87 | 2.52 | 5.00 |
| f__*Ruminococcaceae* | 16.45 | 17.60 | 25.28 |
| g__*Oscillospira* | 1.85 | 0.29 | 2.20 |
| g__*Ruminococcus* | 0.16 | 0.03 | 1.51 |
| g__*unclassified* | 14.44 | 17.28 | 21.50 |
| f__*Veillonellaceae* | 1.53 | 1.44 | 1.24 |
| g__*Phascolarctobacterium* | 1.45 | 1.43 | 1.18 |
| f__unclassified | 6.27 | 6.45 | 5.40 |
| Other | 0.73 | 0.21 | 0.81 |
| p__*Planctomycetes* | 0.78 | 1.32 | 0.58 |
| c__*vadinHA49* | 0.78 | 1.32 | 0.58 |
| o__*PeHg47* | 0.78 | 1.32 | 0.58 |
| f__unclassified | 0.78 | 1.32 | 0.58 |
| p__*Proteobacteria* | 14.39 | 17.05 | 8.21 |
| c__*Betaproteobacteria* | 5.11 | 2.90 | 2.36 |
| o__*Burkholderiales* | 1.04 | 1.27 | 0.34 |
| f__*Alcaligenaceae* | 0.58 | 0.95 | 0.11 |
| g__*Sutterella* | 0.50 | 0.95 | 0.07 |
| o__*Rhodocyclales* | 3.48 | 1.11 | 1.78 |
| f__*Rhodocyclaceae* | 3.48 | 1.11 | 1.78 |
| g__unclassified | 2.33 | 0.38 | 1.15 |
| Other | 1.15 | 0.73 | 0.63 |
| o__unclassified | 0.47 | 0.51 | 0.20 |
| c__*Deltaproteobacteria* | 1.92 | 1.60 | 1.53 |
| o__*Desulfovibrionales* | 1.71 | 1.58 | 1.42 |
| f__*Desulfovibrionaceae* | 1.70 | 1.58 | 1.42 |
| g__unclassified | 1.32 | 1.19 | 1.13 |
| c__*Gammaproteobacteria* | 7.29 | 12.24 | 4.30 |
| o__*Aeromonadales* | 6.19 | 10.97 | 2.99 |
| f__*Succinivibrionaceae* | 6.19 | 10.97 | 2.99 |
| g__*Succinatimonas* | 6.18 | 10.88 | 2.98 |
| o__*Enterobacteriales* | 0.74 | 1.03 | 1.08 |
| f__*Enterobacteriaceae* | 0.74 | 1.03 | 1.08 |
| g__unclassified | 0.60 | 0.92 | 0.90 |
| p__*Synergistetes* | 3.86 | 9.03 | 2.12 |
| c__*Synergistia* | 3.86 | 9.03 | 2.12 |
| o__*Synergistales* | 3.86 | 9.03 | 2.12 |
| f__*Synergistaceae* | 3.86 | 8.95 | 2.12 |
| g__*Synergistes* | 3.70 | 8.70 | 2.05 |
| p__*Verrucomicrobia* | 1.38 | 0.03 | 0.70 |
| c__*Verrucomicrobiae* | 1.33 | 0.03 | 0.56 |
| o__*Verrucomicrobiales* | 1.33 | 0.03 | 0.56 |
| f__*Verrucomicrobiaceae* | 1.33 | 0.03 | 0.56 |
| g__*Akkermansia* | 1.30 | 0.03 | 0.53 |
| p__unclassified | 0.08 | 2.76 | 0.08 |
| k__unmapped | 18.14 | 20.52 | 22.81 |
