# Supplementary material for: Gene and genome-centric analyses of koala and wombat fecal microbiomes point to metabolic specialization for Eucalyptus digestion
Source: PeerJ. 2017 Nov 16;5:e4075. doi: 10.7717/peerj.4075 (PMC5697889; doi:10.7717/peerj.4075)
Supplement: Table S3 — Shotgun reads corresponding to 16S rRNA sequences were identified with HMMs and mapped to the Greengenes 97% database (McDonald et al., 2012), and the resulting community makeup was assessed at each taxonomic level (including unmapped reads). Lineages with less than 0.05% of corresponding reads were grouped and reported as “Other” (if the resulting group represented >0.05% of the community). Abbreviations; k, kingdom; p, phylum; c, class; o, order; f, family; g, genus. [file peerj-05-4075-s006.docx]

| **Taxonomy** | **Wombat_1** | **Wombat_2** | **Wombat_3** | **Wombat_4** | **Wombat_5** |
| --- | --- | --- | --- | --- | --- |
| k__*Archaea* | 3.84 | 2.14 | 1.77 | 0.64 | 1.33 |
| p__*Euryarchaeota* | 3.84 | 2.14 | 1.77 | 0.64 | 1.33 |
| c__*Methanomicrobia* | 3.61 | 2.00 | 1.68 | 0.58 | 1.18 |
| o__*Methanomicrobiales* | 3.61 | 1.89 | 1.68 | 0.58 | 1.15 |
| f__*Methanocorpusculaceae* | 3.13 | 1.60 | 1.40 | 0.48 | 0.99 |
| g__*Methanocorpusculum* | 2.33 | 1.21 | 0.98 | 0.35 | 0.74 |
| g__unclassified | 0.79 | 0.39 | 0.42 | 0.13 | 0.24 |
| k__*Bacteria* | 83.23 | 86.85 | 91.35 | 93.14 | 91.88 |
| p__*Bacteroidetes* | 12.17 | 14.29 | 16.29 | 19.36 | 16.98 |
| c__*Bacteroidia* | 12.05 | 14.22 | 16.24 | 19.30 | 16.94 |
| o__*Bacteroidales* | 12.05 | 14.22 | 16.24 | 19.30 | 16.94 |
| f__*BS11* | 3.43 | 2.78 | 0.22 | 0.24 | 0.36 |
| g__unclassified | 3.43 | 2.78 | 0.22 | 0.24 | 0.36 |
| f__*Bacteroidaceae* | 3.49 | 5.55 | 7.17 | 7.09 | 11.95 |
| g__*Bacteroides* | 3.48 | 5.55 | 7.17 | 7.08 | 11.95 |
| f__*Porphyromonadaceae* | 1.59 | 0.96 | 1.86 | 2.26 | 2.15 |
| g__*Parabacteroides* | 1.57 | 0.94 | 1.82 | 2.26 | 2.12 |
| f__*Prevotellaceae* | 0.12 | 0.21 | 0.48 | 0.99 | 0.21 |
| g__*Prevotella* | 0.11 | 0.20 | 0.42 | 0.80 | 0.20 |
| f__*Rikenellaceae* | 0.74 | 0.93 | 1.27 | 1.06 | 0.42 |
| g__unclassified | 0.49 | 0.59 | 0.63 | 0.42 | 0.26 |
| Other | 0.26 | 0.33 | 0.64 | 0.64 | 0.16 |
| f__[*Paraprevotellaceae*] | 0.50 | 2.08 | 2.21 | 1.28 | 0.47 |
| g__*YRC22* | 0.37 | 1.92 | 1.99 | 1.02 | 0.40 |
| f__*p-2534-18B5* | 0.59 | 0.48 | 0.79 | 1.32 | 0.32 |
| g__unclassified | 0.39 | 0.33 | 0.47 | 0.88 | 0.26 |
| f__unclassified | 1.21 | 0.91 | 1.67 | 4.25 | 0.48 |
| Other | 0.38 | 0.32 | 0.58 | 0.79 | 0.58 |
| p__*Firmicutes* | 52.01 | 57.01 | 53.41 | 50.72 | 61.59 |
| c__*Bacilli* | 0.38 | 0.57 | 0.32 | 0.69 | 0.48 |
| o__*Lactobacillales* | 0.36 | 0.57 | 0.31 | 0.69 | 0.47 |
| f__*Streptococcaceae* | 0.34 | 0.47 | 0.24 | 0.63 | 0.27 |
| g__*Streptococcus* | 0.32 | 0.47 | 0.24 | 0.63 | 0.27 |
| c__*Clostridia* | 51.31 | 56.15 | 52.77 | 49.51 | 60.67 |
| o__*Clostridiales* | 51.25 | 56.10 | 52.72 | 49.47 | 60.63 |
| f__*Christensenellaceae* | 10.01 | 11.26 | 10.54 | 7.50 | 12.92 |
| g__unclassified | 10.01 | 11.24 | 10.54 | 7.50 | 12.92 |
| f__*Clostridiaceae* | 1.83 | 2.27 | 1.38 | 1.92 | 1.99 |
| g__*Clostridium* | 0.78 | 0.94 | 0.80 | 0.79 | 0.92 |
| g__unclassified | 0.72 | 0.90 | 0.38 | 0.75 | 0.76 |
| f__*Lachnospiraceae* | 3.53 | 5.48 | 4.77 | 5.74 | 7.42 |
| g__*Blautia* | 0.13 | 0.30 | 0.31 | 0.80 | 0.86 |
| g__*Coprococcus* | 0.92 | 1.27 | 1.52 | 1.40 | 1.74 |
| g__unclassified | 1.89 | 3.24 | 2.40 | 2.67 | 3.65 |
| Other | 0.59 | 0.66 | 0.54 | 0.87 | 1.17 |
| f__*Ruminococcaceae* | 22.25 | 24.22 | 19.22 | 18.11 | 22.73 |
| g__*Oscillospira* | 1.51 | 2.02 | 1.71 | 2.52 | 2.77 |
| g__*Ruminococcus* | 6.55 | 7.16 | 4.95 | 2.68 | 4.68 |
| g__unclassified | 14.02 | 14.82 | 12.29 | 12.54 | 14.91 |
| f__*Veillonellaceae* | 1.39 | 1.58 | 2.08 | 5.69 | 2.75 |
| g__*Acidaminococcus* | 0.24 | 0.27 | 0.51 | 1.03 | 0.42 |
| g__*Megasphaera* | 0.32 | 0.23 | 0.56 | 1.69 | 0.90 |
| g__*Phascolarctobacterium* | 0.24 | 0.48 | 0.84 | 2.80 | 1.23 |
| g__unclassified | 0.56 | 0.56 | 0.11 | 0.09 | 0.16 |
| f__[*Mogibacteriaceae*] | 0.48 | 0.32 | 0.26 | 0.66 | 0.22 |
| g__unclassified | 0.45 | 0.30 | 0.23 | 0.62 | 0.21 |
| f__unclassified | 11.48 | 10.57 | 14.18 | 9.40 | 12.24 |
| c__*Erysipelotrichi* | 0.30 | 0.25 | 0.28 | 0.50 | 0.42 |
| o__*Erysipelotrichales* | 0.30 | 0.25 | 0.28 | 0.50 | 0.42 |
| f__*Erysipelotrichaceae* | 0.30 | 0.25 | 0.28 | 0.50 | 0.42 |
| Other | 0.30 | 0.25 | 0.28 | 0.50 | 0.42 |
| p__*Lentisphaerae* | 0.89 | 0.41 | 0.39 | 0.13 | 0.15 |
| c__[*Lentisphaeria*] | 0.89 | 0.41 | 0.39 | 0.13 | 0.15 |
| o__*Victivallales* | 0.89 | 0.41 | 0.39 | 0.13 | 0.15 |
| f__*Victivallaceae* | 0.89 | 0.41 | 0.39 | 0.13 | 0.15 |
| g__unclassified | 0.87 | 0.40 | 0.38 | 0.12 | 0.13 |
| p__*Proteobacteria* | 4.68 | 4.15 | 4.93 | 5.13 | 6.43 |
| c__*Alphaproteobacteria* | 0.15 | 0.07 | 0.43 | 0.14 | 0.95 |
| o__*Rhodospirillales* | 0.09 | 0.04 | 0.30 | 0.07 | 0.60 |
| f__*Rhodospirillaceae* | 0.09 | 0.04 | 0.30 | 0.07 | 0.60 |
| Other | 0.09 | 0.04 | 0.30 | 0.07 | 0.60 |
| c__*Betaproteobacteria* | 0.24 | 0.27 | 0.32 | 0.47 | 0.61 |
| o__*Burkholderiales* | 0.22 | 0.26 | 0.31 | 0.47 | 0.60 |
| f__*Alcaligenaceae* | 0.13 | 0.15 | 0.18 | 0.19 | 0.54 |
| g__*Sutterella* | 0.13 | 0.14 | 0.18 | 0.19 | 0.54 |
| c__*Deltaproteobacteria* | 1.12 | 0.75 | 1.48 | 1.73 | 0.53 |
| o__*Desulfovibrionales* | 1.12 | 0.75 | 1.48 | 1.73 | 0.53 |
| f__*Desulfovibrionaceae* | 1.12 | 0.75 | 1.48 | 1.73 | 0.53 |
| g__*Bilophila* | 0.87 | 0.47 | 0.98 | 0.82 | 0.17 |
| g__*Desulfovibrio* | 0.20 | 0.19 | 0.41 | 0.79 | 0.29 |
| c__*Gammaproteobacteria* | 3.09 | 3.04 | 2.63 | 2.74 | 4.07 |
| o__*Aeromonadales* | 3.01 | 2.95 | 2.60 | 2.72 | 3.36 |
| f__*Succinivibrionaceae* | 3.01 | 2.95 | 2.60 | 2.72 | 3.36 |
| g__*Succinivibrio* | 2.61 | 2.66 | 2.32 | 2.40 | 3.00 |
| o__*Enterobacteriales* | 0.06 | 0.08 | 0.01 | 0.02 | 0.69 |
| f__*Enterobacteriaceae* | 0.06 | 0.08 | 0.01 | 0.02 | 0.69 |
| g__unclassified | 0.06 | 0.07 | 0.01 | 0.01 | 0.59 |
| p__*Spirochaetes* | 4.49 | 2.78 | 3.35 | 2.79 | 1.24 |
| c__*Spirochaetes* | 4.49 | 2.78 | 3.35 | 2.79 | 1.24 |
| o__*Sphaerochaetales* | 2.36 | 1.21 | 1.70 | 1.59 | 0.47 |
| f__*Sphaerochaetaceae* | 2.36 | 1.21 | 1.70 | 1.59 | 0.47 |
| g__*Sphaerochaeta* | 2.35 | 1.21 | 1.70 | 1.59 | 0.47 |
| o__*Spirochaetales* | 2.04 | 1.52 | 1.58 | 1.17 | 0.74 |
| f__*Spirochaetaceae* | 2.04 | 1.52 | 1.58 | 1.17 | 0.74 |
| g__*Treponema* | 2.04 | 1.52 | 1.57 | 1.16 | 0.74 |
| p__*Synergistetes* | 1.36 | 1.50 | 2.18 | 1.29 | 0.72 |
| c__*Synergistia* | 1.36 | 1.50 | 2.18 | 1.29 | 0.72 |
| o__*Synergistales* | 1.36 | 1.50 | 2.18 | 1.29 | 0.72 |
| f__*Synergistaceae* | 1.33 | 1.44 | 2.18 | 1.29 | 0.72 |
| g__*Synergistes* | 1.25 | 1.38 | 2.12 | 1.25 | 0.69 |
| p__*Tenericutes* | 5.69 | 4.80 | 6.75 | 8.78 | 2.36 |
| c__*Mollicutes* | 4.93 | 4.59 | 6.40 | 8.49 | 2.30 |
| o__*RF39* | 4.93 | 4.59 | 6.40 | 8.49 | 2.30 |
| f__unclassified | 4.93 | 4.59 | 6.40 | 8.49 | 2.30 |
| c__*RF3* | 0.75 | 0.21 | 0.35 | 0.29 | 0.06 |
| o__*ML615J-28* | 0.75 | 0.21 | 0.35 | 0.29 | 0.06 |
| f__unclassified | 0.75 | 0.21 | 0.35 | 0.29 | 0.06 |
| p__*Verrucomicrobia* | 1.47 | 1.40 | 3.50 | 4.30 | 1.99 |
| c__*Verrucomicrobiae* | 1.42 | 1.35 | 3.44 | 4.21 | 1.87 |
| o__*Verrucomicrobiales* | 1.42 | 1.35 | 3.44 | 4.21 | 1.87 |
| f__*Verrucomicrobiaceae* | 1.42 | 1.35 | 3.44 | 4.21 | 1.87 |
| g__*Akkermansia* | 1.42 | 1.35 | 3.43 | 4.21 | 1.86 |
| Other | 0.47 | 0.51 | 0.56 | 0.64 | 0.43 |
| k__unmapped | 12.93 | 11.02 | 6.89 | 6.22 | 6.79 |
