# Supplementary material for: Gene and genome-centric analyses of koala and wombat fecal microbiomes point to metabolic specialization for Eucalyptus digestion
Source: PeerJ. 2017 Nov 16;5:e4075. doi: 10.7717/peerj.4075 (PMC5697889; doi:10.7717/peerj.4075)
Supplement: Table S4 — Reads mapping to 16S and 18S rRNA genes were extracted from quality-filtered shotgun sequencing samples using hidden Markov models and mapped to the SILVA 98% database (Quast et al., 2013). Table values are kingdom-level percentages of all extracted reads. Here, lca is last common ancestor. [file peerj-05-4075-s007.docx]

|  | **k_*Archaea*** | **k_*Bacteria*** | **k_*Eukaryota*** | **k_unmapped** | **k_unresolved_by_lca** |
| --- | --- | --- | --- | --- | --- |
| Koala_1 | 0.0227% | 82.2% | 0.0363% | 17.8% | 0.00450% |
| Koala_2 | 0.0350% | 80.4% | 0.131% | 19.4% | 0.0262% |
| Koala_3 | 0.351% | 81.0% | 0% | 18.6% | 0.00950% |
| Wombat_1 | 1.92% | 91.5% | 0.0278% | 6.60% | 0% |
| Wombat_2 | 3.98% | 84.1% | 0.0582% | 11.9% | 0% |
| Wombat_3 | 1.41% | 92.4% | 0.239% | 5.97% | 0% |
| Wombat_4 | 2.22% | 87.9% | 0.0285% | 9.90% | 0% |
| Wombat_5 | 0.659% | 93.6% | 0.0276% | 5.75% | 0% |
