# Supplementary material for: Gene and genome-centric analyses of koala and wombat fecal microbiomes point to metabolic specialization for Eucalyptus digestion
Source: PeerJ. 2017 Nov 16;5:e4075. doi: 10.7717/peerj.4075 (PMC5697889; doi:10.7717/peerj.4075)
Supplement: Table S5 — All time-points from each host dataset were co-assembled de novo (CLC) following stringent quality control of raw reads (adaptor trimming with SeqPrep and quality trimming with Nesoni). Co-assemblies were then validated by mapping high-quality paired reads from each time-point back to the corresponding assembly (BWA). [file peerj-05-4075-s008.docx]

|  | **Total length**  **(with scaffolds)** | **Contigs** | **N25** | **N50** | **Maximum contig length** | **QC pairs mapped to assembly** |
| --- | --- | --- | --- | --- | --- | --- |
| Wombat | 686.7 Mbp | 723,167 | 21,575 bp | 2,451 bp | 693,555 bp | 76.5 – 84.9% |
| Koala | 305.2 Mbp | 292,692 | 24,061 bp | 2,611 bp | 951,760 bp | 87.2 – 90.4% |
