# Supplementary material for: Gene and genome-centric analyses of koala and wombat fecal microbiomes point to metabolic specialization for Eucalyptus digestion
Source: PeerJ. 2017 Nov 16;5:e4075. doi: 10.7717/peerj.4075 (PMC5697889; doi:10.7717/peerj.4075)
Supplement: Table S6 [file peerj-05-4075-s009.docx]

| **ID^a^** | **Taxonomy^b^** | | **Bin size**  **(Mb)** | **Completeness**  **(%)^c^** | **Contamination**  **(%)^c^** | **GC**  **(%)** | **% Community^d^** | | |
| --- | --- | --- | --- | --- | --- | --- | --- | --- | --- |
|  |  |  |  |  |  |  | **(Median)** | **(Mean)** | |
| k01 | k_*Bacteria*; p_*Bacteroidetes*; c_*Bacteroidia*; o_*Bacteroidales*; f_*S24-7* | | 4.46 | 95.5 | 0.9 | 51.3 | 7.78 | 6.69 | |
| k02 | k_*Bacteria*; p_*Firmicutes*; c_*Clostridia*; o_*Clostridiales*; f_*Lachnospiraceae* | | 3.34 | 98.7 | 0.8 | 52.8 | 4.16 | 4.34 | |
| k03 | k_*Bacteria*; p_*Firmicutes*; c_*Clostridia*; o_*Clostridiales*; f_*Lachnospiraceae* | | 1.61 | 81.5 | 6.2 | 55.8 | 4.16 | 3.87 | |
| k04 | k_*Bacteria*; p_*Firmicutes*; c_*Clostridia*; o_*Clostridiales*; f_*Oscillospiraceae* | | 2.36 | 86.6 | 9.8 | 60.6 | 3.75 | 3.63 | |
| k05 | k_*Bacteria*; p_*Proteobacteria*; c_*Betaproteobacteria*; o_*Rhodocyclales*; f_*Rhodocyclaceae* | | 2.83 | 67.0 | 6.5 | 56.5 | 2.30 | 2.31 | |
| k06 | k_*Bacteria*; p_*Firmicutes*; c_*Clostridia*; o_*Clostridiales*; f_*Oscillospiraceae* | | 1.98 | 67.2 | 0.7 | 58.4 | 2.12 | 2.65 | |
| k07 | k_*Bacteria*; p_*Firmicutes*; c_*Clostridia*; o_*Clostridiales*; f_*Ruminococcaceae*; g_*Ruminococcus* | | 2.08 | 68.5 | 4.9 | 47.0 | 1.52 | 1.21 | |
| k08 | k_*Bacteria*; p_*Bacteroidetes*; c_*Bacteroidia*; o_*Bacteroidales*; f_*Rikenellaceae*; g_*Alistipes* | | 3.51 | 91.5 | 0.0 | 58.8 | 1.27 | 1.06 | |
| k09 | k_*Bacteria*; p_*Synergistetes*; c_*Synergistia*; o_*Synergistales*; f_*Synergistaceae* | | 3.44 | 99.4 | 3.4 | 55.3 | 1.26 | 1.60 | |
| k10 | k_*Bacteria*; p_*Bacteroidetes*; c_*Bacteroidia*; o_*Bacteroidales*; f_*S24-7* | | 2.75 | 98.8 | 1.1 | 51.2 | 1.23 | 0.94 | |
| k11 | k_*Bacteria*; p_*Firmicutes*; c_*Clostridia*; o_*Clostridiales*; f_*Ruminococcaceae*; g_*Ruminococcus* | | 2.00 | 94.3 | 0.0 | 45.1 | 1.02 | 1.90 | |
| k12 | k_*Bacteria*; p_*Firmicutes*; c_*Clostridia*; o_*Clostridiales*; f_*Oscillospiraceae* | | 1.90 | 78.6 | 0.0 | 45.8 | 0.27 | 0.32 | |
| k13 | k_*Bacteria*; p_*Firmicutes*; c_*Clostridia*; o_*Clostridiales*; f_*Lachnospiraceae* | | 3.30 | 90.5 | 10.1 | 49.5 | 0.25 | 0.28 | |
| k14 | k_*Bacteria*; p_*Proteobacteria*; c_*Deltaproteobacteria*; o_*Desulfovibrionales*; f_*Desulfovibrionaceae*; g_*Desulfovibrio* | | 2.68 | 53.5 | 7.5 | 60.6 | 0.21 | 0.22 | |
| k15 | k_*Bacteria*; p_*Cyanobacteria*; c_*4C0d-2*; o_*YS2* | | 1.74 | 69.4 | 0.0 | 34.7 | 0.00 | 0.19 | |
|  |  | |  |  | Total: | | 31.19 | | 31.30 |
|  | | | | | | | | | |
| w01 | k_*Bacteria*; p_*Firmicutes*; c_*Clostridia*; o_*Christensenellales*; f_*Christensenellaceae* | 2.85 | | 98.4 | 0.0 | 52.2 | 7.10 | 7.16 | |
| w02 | k_*Bacteria*; p_*Tenericutes*; c_*Mollicutes*; o_*RF39* | 1.57 | | 93.3 | 9.4 | 25.8 | 4.20 | 5.49 | |
| w03 | k_*Bacteria*; p_*Spirochaetes*; c_*Spirochaetes*; o_*Spirochaetales*; f_*Spirochaetaceae*; g_*Sphaerochaeta* | 2.36 | | 90.2 | 2.2 | 48.1 | 3.41 | 2.84 | |
| w04 | k_*Archaea*; p_*Euryarchaeota*; c_*Methanomicrobia*; o_*Methanomicrobiales*; f_*Methanocorpusculaceae* | | 1.97 | 91.6 | 2.3 | 52.8 | 1.81 | 2.08 | |
| w05 | k_*Bacteria*; p_*Firmicutes*; c_*Clostridia*; o_*Lachnospirales* | 1.04 | | 62.4 | 1.8 | 28.9 | 1.48 | 1.58 | |
| w06 | k_*Bacteria*; p_*Bacteroidetes*; c_*Bacteroidia*; o_*Bacteroidales*; f_*BS11* | 2.59 | | 97.6 | 0.3 | 38.0 | 1.37 | 3.64 | |
| w07 | k_*Bacteria*; p_*Firmicutes*; c_*Clostridia*; o_*Christensenellales*; f_*Christensenellaceae* | 2.21 | | 96.7 | 0.8 | 46.2 | 1.26 | 1.44 | |
| w08 | k_*Bacteria*; p_*Verrucomicrobia*; c_*Verrucomicrobiae*; o_*Verrucomicrobiales*; f_*Verrucomicrobiaceae*; g_*Akkermansia* | 2.44 | | 88.6 | 0.7 | 55.3 | 1.25 | 1.01 | |
| w09 | k_*Bacteria*; p_*Proteobacteria*; c_*Gammaproteobacteria*; o_*Aeromonadales*; f_*Succinivibrionaceae*; g_*Succinivibrio* | 2.31 | | 93.1 | 0.3 | 36.8 | 1.15 | 1.20 | |
| w10 | k_*Bacteria*; p_*Firmicutes*; c_*Clostridia*; o_*Christensenellales*; f_*Christensenellaceae* | 1.02 | | 68.3 | 1.5 | 46.7 | 0.79 | 0.84 | |
| w11 | k_*Bacteria*; p_*Firmicutes*; c_*Clostridia*; o_*Clostridiales*; f_*Ruminococcaceae*; g_*Ruminococcus* | 3.71 | | 98.7 | 0.0 | 54.8 | 0.71 | 1.20 | |
| w12 | k_*Bacteria*; p_*Bacteroidetes*; c_*Bacteroidia*; o_*Bacteroidales*; f_*BS11* | 2.96 | | 98.4 | 2.4 | 37.5 | 0.66 | 0.91 | |
| w13 | k_*Bacteria*; p_*Bacteroidetes*; c_*Bacteroidia*; o_*Bacteroidales*; f_*Bacteroidaceae*; g_*Bacteroides* | 5.83 | | 95.4 | 3.6 | 42.4 | 0.55 | 0.64 | |
| w14 | k_*Bacteria*; p_*Tenericutes*; c_*Mollicutes* | 1.69 | | 92.1 | 0.0 | 28.5 | 0.54 | 0.49 | |
| w15 | k_*Bacteria*; p_*Firmicutes*; c_*Clostridia*; o_*Clostridiales*; f_*Ruminococcaceae*; g_*Ruminococcus* | 2.08 | | 93.3 | 1.0 | 44.7 | 0.52 | 0.44 | |
| w16 | k_*Bacteria*; p_*Firmicutes*; c_*Clostridia*; o_*Clostridiales* | 1.64 | | 96.5 | 0.9 | 39.3 | 0.44 | 0.51 | |
| w17 | k_*Bacteria*; p_*Firmicutes*; c_*Clostridia*; o_*Clostridiales* | 2.62 | | 86.9 | 0.8 | 59.1 | 0.35 | 0.40 | |
| w18 | k_*Bacteria*; p_*Firmicutes*; c_*Clostridia*; o_*Clostridiales* | 1.61 | | 66.5 | 0.7 | 27.3 | 0.43 | 0.34 | |
| w19 | k_*Bacteria*; p_*Bacteroidetes*; c_*Bacteroidia*; o_*Bacteroidales* | 3.62 | | 94.5 | 1.9 | 51.0 | 1.06 | 0.29 | |
| w20 | k_*Bacteria*; p_*Spirochaetes*; c_*Spirochaetia*; o_*Spirochaetales*; f_*Spirochaetaceae*; g_*Treponema* | 2.50 | | 96.1 | 0.7 | 31.8 | 0.32 | 0.27 | |
| w21 | k_*Bacteria*; p_*Bacteroidetes*; c_*Bacteroidia*; o_*Bacteroidales* | 2.37 | | 93.5 | 5.0 | 48.9 | 0.25 | 0.19 | |
| w22 | k_*Bacteria*; p_*Lentisphaerae*; c_*Lentisphaeria*; o_*Victivallales* | 2.20 | | 88.8 | 1.3 | 57.8 | 0.24 | 0.18 | |
| w23 | k_*Bacteria*; p_*Firmicutes*; c_*Clostridia*; o_*Clostridiales* | 2.89 | | 65.4 | 9.4 | 61.6 | 0.21 | 0.17 | |
| w24 | k_*Bacteria*; p_*Firmicutes*; c_*Clostridia*; o_*Clostridiales*; f_*Clostridiaceae*; g_*Clostridium* | 1.98 | | 73.0 | 1.5 | 27.4 | 0.12 | 0.14 | |
| w25 | k_*Bacteria*; p_*Firmicutes*; c_*Clostridia*; o_*Clostridiales* | 2.56 | | 60.7 | 8.0 | 52.5 | 0.16 | 0.13 | |
| w26 | k_*Bacteria*; p_*Firmicutes*; c_*Clostridia*; o_*Clostridiales*; f_*Lachnospiraceae* | 2.79 | | 91.7 | 0.6 | 39.0 | 0.35 | 0.11 | |
| w27 | k_*Bacteria*; p_*Firmicutes*; c_*Clostridia*; o_*Clostridiales* | 2.00 | | 71.9 | 1.7 | 62.5 | 0.27 | 0.11 | |
| w28 | k_*Bacteria*; p_*Firmicutes*; c_*Clostridia*; o_*Clostridiales*; f_*Ruminococcaceae* | 1.60 | | 64.1 | 1.9 | 42.7 | 0.13 | 0.11 | |
| w29 | k_*Bacteria*; p_*Firmicutes*; c_*Clostridia*; o_*Clostridiales*; f_*Lachnospiraceae* | 2.94 | | 95.9 | 0.2 | 35.0 | 0.26 | 0.10 | |
| w30 | k_*Bacteria*; p_*Firmicutes*; c_*Clostridia*; o_*Clostridiales* | 3.19 | | 82.6 | 5.9 | 54.3 | 0.30 | 0.07 | |
| w31 | k_*Bacteria*; p_*Tenericutes*; c_*Mollicutes*; o_*RF39* | 0.85 | | 70.9 | 0.0 | 24.0 | 0.13 | 0.07 | |
| w32 | k_*Bacteria*; p_*Firmicutes*; c_*Clostridia*; o_*Clostridiales*; f_*Lachnospiraceae* | 3.71 | | 69.7 | 5.2 | 38.4 | 0.11 | 0.07 | |
| w33 | k_*Bacteria*; p_*Bacteroidetes*; c_*Bacteroidia*; o_*Bacteroidales* | 3.72 | | 88.6 | 9.4 | 49.1 | 0.14 | 0.06 | |
| w34 | k_*Bacteria*; p_*Proteobacteria*; c_*Deltaproteobacteria*; o_*Desulfovibrionales*; f_*Desulfovibrionaceae* | 2.79 | | 61.6 | 2.5 | 59.0 | 0.12 | 0.02 | |
| w35 | k_*Bacteria*; p_*Firmicutes*; c_*Clostridia*; o_*Clostridiales* | 1.61 | | 60.0 | 1.4 | 49.1 | 0.10 | 0.02 | |
| w36 | k_*Archaea*; p_*Thermoplasmata* | 3.30 | | 65.2 | 6.0 | 56.2 | 0.07 | 0.01 | |
| w37 | k_*Bacteria*; p_*Firmicutes*; c_*Clostridia*; o_*Clostridiales* | 1.61 | | 62.8 | 2.3 | 27.9 | 0.14 | 0.00 | |
| w38 | k_*Bacteria*; p_*Firmicutes*; c_*Clostridia*; o_*Clostridiales* | 1.60 | | 63.8 | 2.1 | 30.8 | 0.11 | 0.00 | |
|  |  |  | |  | Total: | | 36.86 | 30.10 | |

^a^ Named for host (**k**oala or **w**ombat) and rank based on median abundance.

^b^ Assigned based on alignment of kingdom-specific (bacterial or archaeal) concatenated marker genes alongside RefSeq reference genomes.

^c^ Calculated based on lineage-specific single-copy marker genes.

^d^ Calculated based on average read coverage of population genome contigs across time-points.
